# Morphological Analysis of the M1 Segment of the Middle Cerebral Artery and Its Implications for Neurosurgical Approaches: A Cadaveric Study

**DOI:** 10.7759/cureus.107780

**Published:** 2026-04-27

**Authors:** Harsimran Jit Singh, Anjali Aggarwal, Anjali Singal, Sushant S Das, Kanav Gupta, Daisy Sahni

**Affiliations:** 1 Anatomy, All India Institute of Medical Sciences, Vijaypur, Jammu, Jammu, IND; 2 Anatomy, Postgraduate Institute of Medical Education and Research, Chandigarh, Chandigarh, IND; 3 Anatomy, All India Institute of Medical Sciences, Bathinda, Bathinda, IND; 4 Neurosurgery, All India Institute of Medical Sciences, Vijaypur, Jammu, Jammu, IND; 5 Anatomy, Shri Mata Vaishno Devi Institute of Medical Excellence, Jammu, IND

**Keywords:** anatomical variations, cadaveric study, cerebrovascular surgery, lenticulostriate perforators, m1 segment, middle cerebral artery, neurosurgical approaches

## Abstract

Anatomical variations of the middle cerebral artery (MCA) and its lenticulostriate perforator branches can confound imaging interpretation and increase ischemic injury risk during various procedures, particularly around the M1 segment, where aneurysms arise. This cadaveric study characterized M1 segment morphology and perforator distribution. Thirty formalin-fixed cerebral hemispheres (27 males and three females) with a mean age of 68 years (ranging from 48 years to 77 years) were dissected under 2.5× magnification with arachnoid removal; M1 origin relative to optic nerve and chiasma were documented, M1 diameter and length were measured, division patterns were recorded, and perforators were quantified on M1 segment of MCA and classified as direct or indirect. M1 length ranged from 8.42 to 24.17 mm (mean 17.13 ± 3.4 mm), and the origin diameter was 2.35 ± 0.51 mm. The MCA originated lateral to the optic chiasma in 25 (83%) and lateral to the optic nerve in five (17%). True bifurcation predominated in 23 (76.7%), with pseudotrifurcation in three (10%), true trifurcation in two (6.6%), and early bifurcation in two (6.6%). Perforator counts varied (0-9), with the highest in the intermediate direct group (mean 4). Forty-seven post-bifurcation perforators were identified, all from the upper division of MCA. Early bifurcation cases lacked medial perforators and showed denser intermediate and post-bifurcation perforation. These findings support identifying perforator-free zones, avoiding perforator injury during clipping, and incorporating cerebral vascular cadaveric dissection into neurosurgical training.

## Introduction

Anatomic variations have been documented in the literature and are frequently identified as tangential findings. The prevalence and nature of these variations are influenced by the specific population, as most of them are governed by various genetic factors. Although they are mostly asymptomatic, these variations pose challenges in clinical settings [1]. The middle cerebral artery (MCA) is considered the most vital and complex cerebral vessel, supplying blood to major cortical and deeper areas of the brain. Direct surgical approaches to the proximal MCA require brain retraction and microsurgical dissection, which may cause ischemia during vessel manipulation [2]. Small lenticulostriate arteries (LSAs), also known as perforator arteries, arise from the MCA. They supply blood to crucial subcortical areas, including the basal ganglia and posterior limb of the internal capsule [3-5]. The occlusion or stenosis of the LSA may lead to small infarcts or strokes, which should be differentiated from large-artery atherosclerosis when determining the cause of the particular stroke [6,7]. Establishing a perforator-free zone before clipping is essential to avoid severe neurological impairment [8,9]. The present study focuses on the anatomical variations of the MCA, particularly the M1 segment, and highlights the importance of understanding the morphometry, branching patterns, and perforator distribution of M1 to avoid neurological damage during surgery. Addressing gaps in existing research, the study offers a detailed cadaveric analysis combining topographical and morphological classifications of perforators and examines their relationship with M1 division variations, such as early bifurcation. It is worth noting that incorporating cadaveric dissection of the cerebral vasculature into the neurosurgical training curriculum would enable neurosurgical residents to grasp the intricate neuroanatomy of the brain vasculature and build confidence during surgical procedures [10-13].

## Materials and methods

Thirty normal, formalin-fixed cerebral hemispheres (27 males and three females) with a mean age of 68 years (ranging from 48 years to 77 years) were obtained from cadavers through the voluntary body donation program of the Department of Anatomy at the Postgraduate Institute of Medical Education and Research, PGIMER, Chandigarh, India. The specimens with visible pathology, deformities, or prior surgical alterations were excluded from the study. The specimens were thoroughly rinsed with water to remove excess formalin. Each brain was examined under a magnoscope (2.5× magnification). The arachnoid mater was carefully removed using fine forceps to expose the Sylvian fissure. The superficial veins were meticulously dissected to reveal the MCA trunk and its branches. The temporal lobe was gently retracted to obtain a complete view of the origin and course of the MCA. Various anatomical parameters of the M1 segment of the MCA were recorded for each of the hemispheres. The relationship between the origin of the MCA from the internal carotid artery (ICA), the optic chiasma, and the optic nerve was carefully documented. The external diameter of the MCA at its origin was measured using a digital caliper (Mitutoyo, Tokyo, Japan). The total length of the M1 segment from its origin to the bifurcation point was measured using a digital caliper. The MCA is divided into two main branches: superior and inferior divisions.

Perforator analysis

Total Number of Perforators

The total number of perforating arteries originating from the M1 segment was recorded for each hemisphere.

Arrangement of Perforators

The spatial distribution and pattern of perforators arising from the M1 segment were documented.

The study was conducted in full compliance with institutional ethical guidelines and applicable regulations of the body donation program.

## Results

Main trunk (MT) of the MCA

The MCA was identified and traced from its origin, starting from the ICA. Its arterial course was further traced along the lateral fissure in the present study. A false bifurcation/trifurcation pattern of the MT of MCA was also examined. The total length of the MT was assessed from the origin to the bifurcation or trifurcation points.

The M1 segment length ranged from 08.42 mm to 24.17 mm, with a mean length of 17.13 ± 3.4 mm. No significant differences were observed between the left and the right cerebral hemispheres. The outer diameter of the M1 segment of the MCA near the origin was 2.35 ± 0.51 mm, ranging from 3.05 to 1.96 mm.

**Figure 1:**
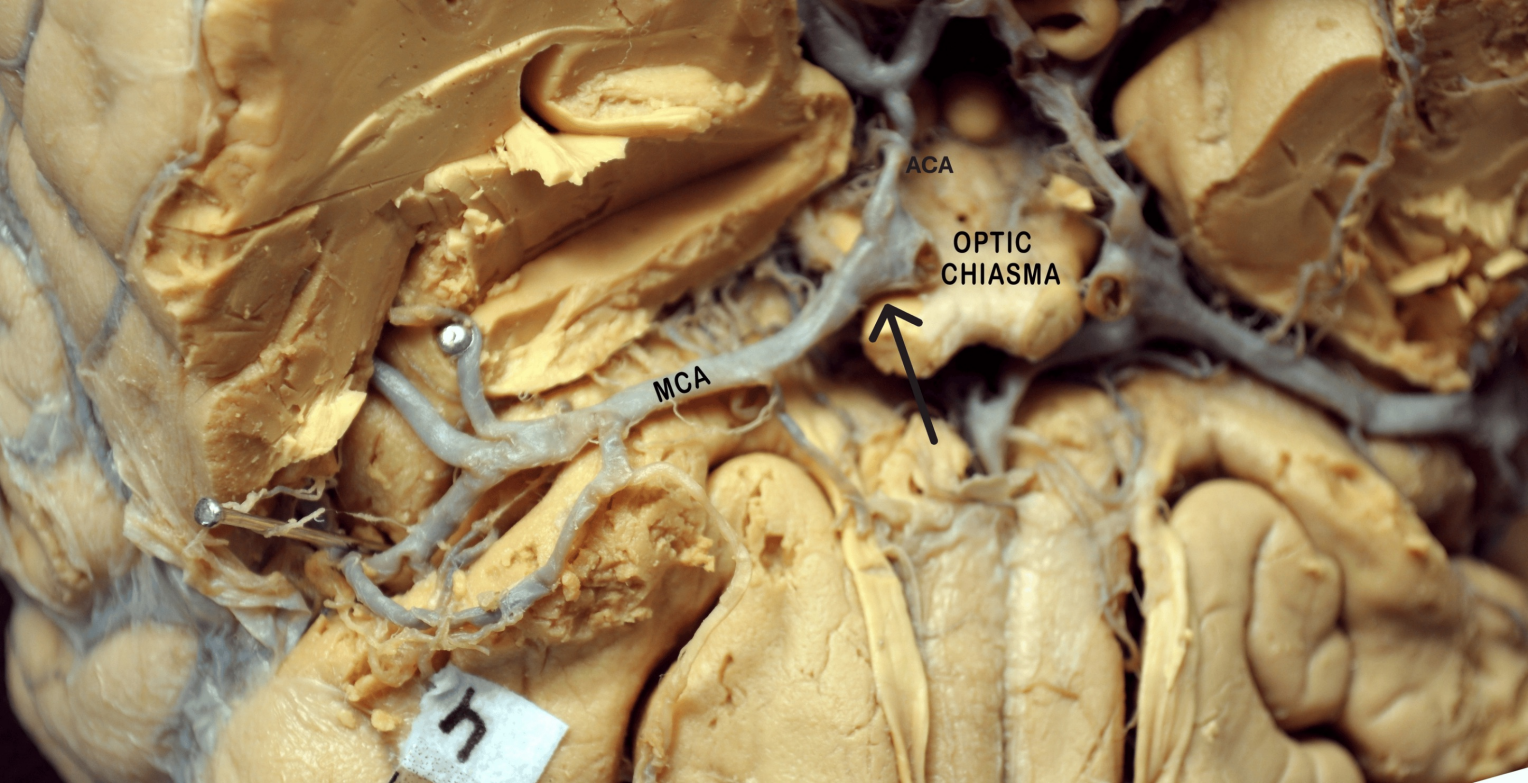
Basal surface of the brain showing MCA's origin is lying exactly opposite the optic Chiasma. The black arrow indicates the origin of MCA. MCA: middle cerebral artery; ACA: anterior cerebral artery

**Figure 2:**
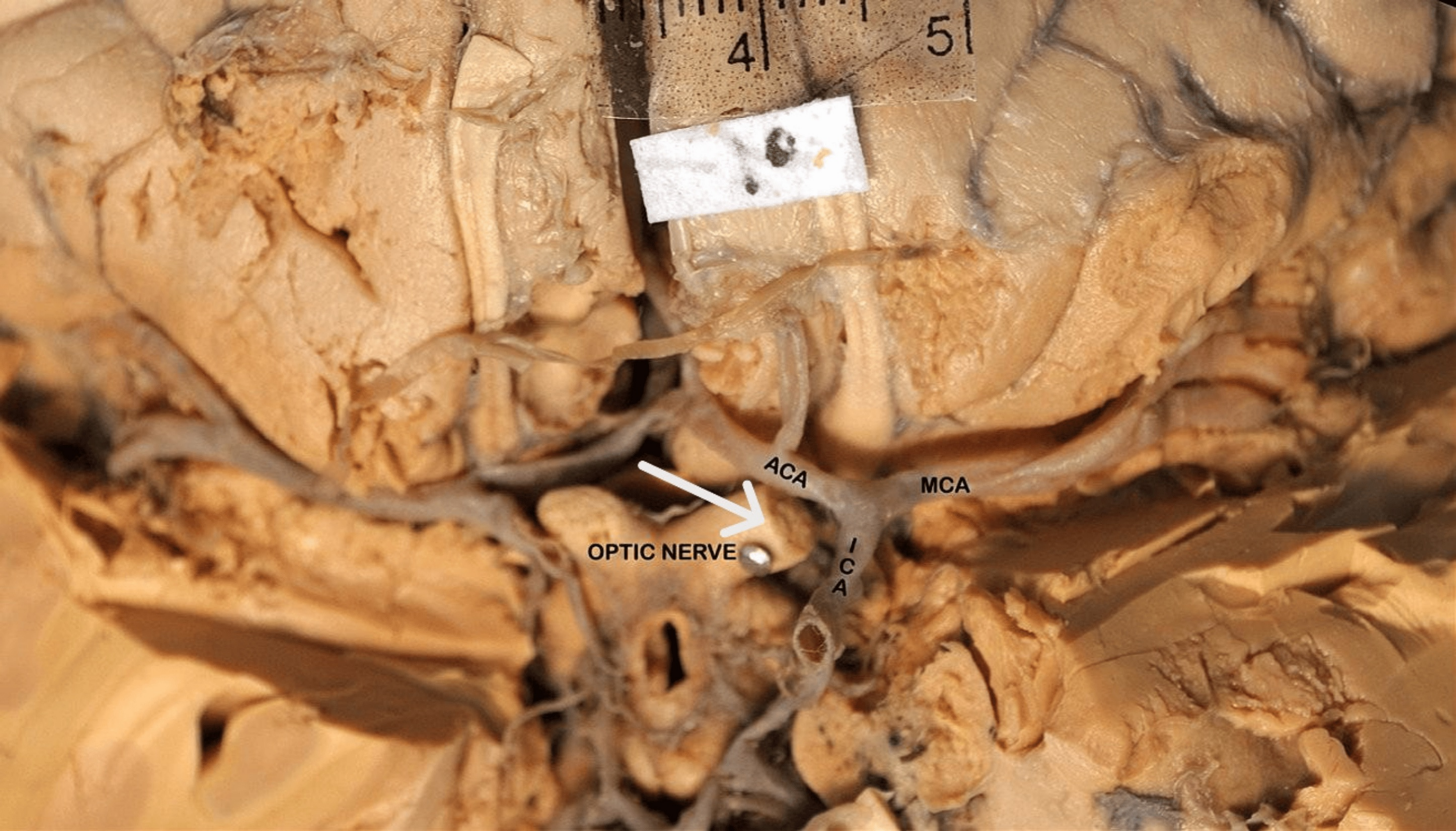
Basal surface of the brain showing the division of internal carotid artery (ICA) into the anterior cerebral artery (ACA) and middle cerebral artery (MCA). The white arrow indicates the optic nerve. This diagram showing the MCA origin is exactly opposite the optic nerve.

In 25 (83%) hemispheres, the origin of the MCA was lateral to the optic chiasma (Figure 1), whereas in five (17%) cases, it was lateral to the optic nerve (Figure 2 and Table 1).

**Table 1:** Relationship between the origin of the middle cerebral artery (MCA) and the optic chiasm and optic nerve.

Lateral to the optic chiasma		Lateral to the optic nerve	
Number	Percentage	Number	Percentage
25	83	5	17

**Figure 3:**
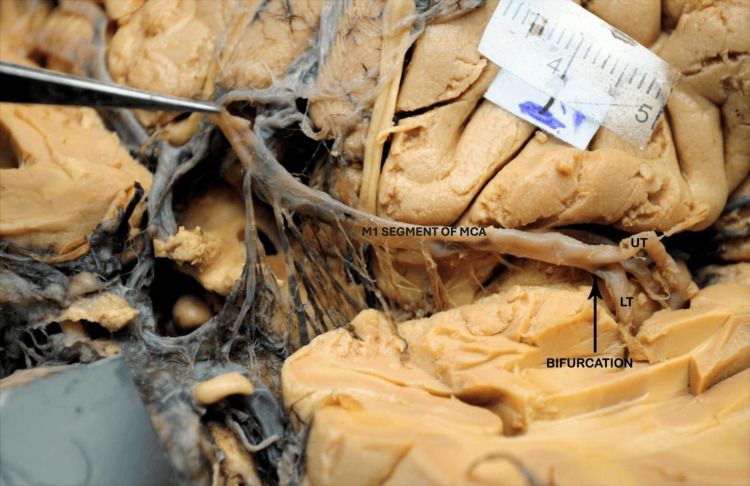
Basal surface of brain showing the bifurcation of the M1 segment of MCA into UT and LT. The black arrow indicates the exact site of bifurcation. MCA: middle cerebral artery; UT: upper trunk; LT: lower trunk

**Figure 4:**
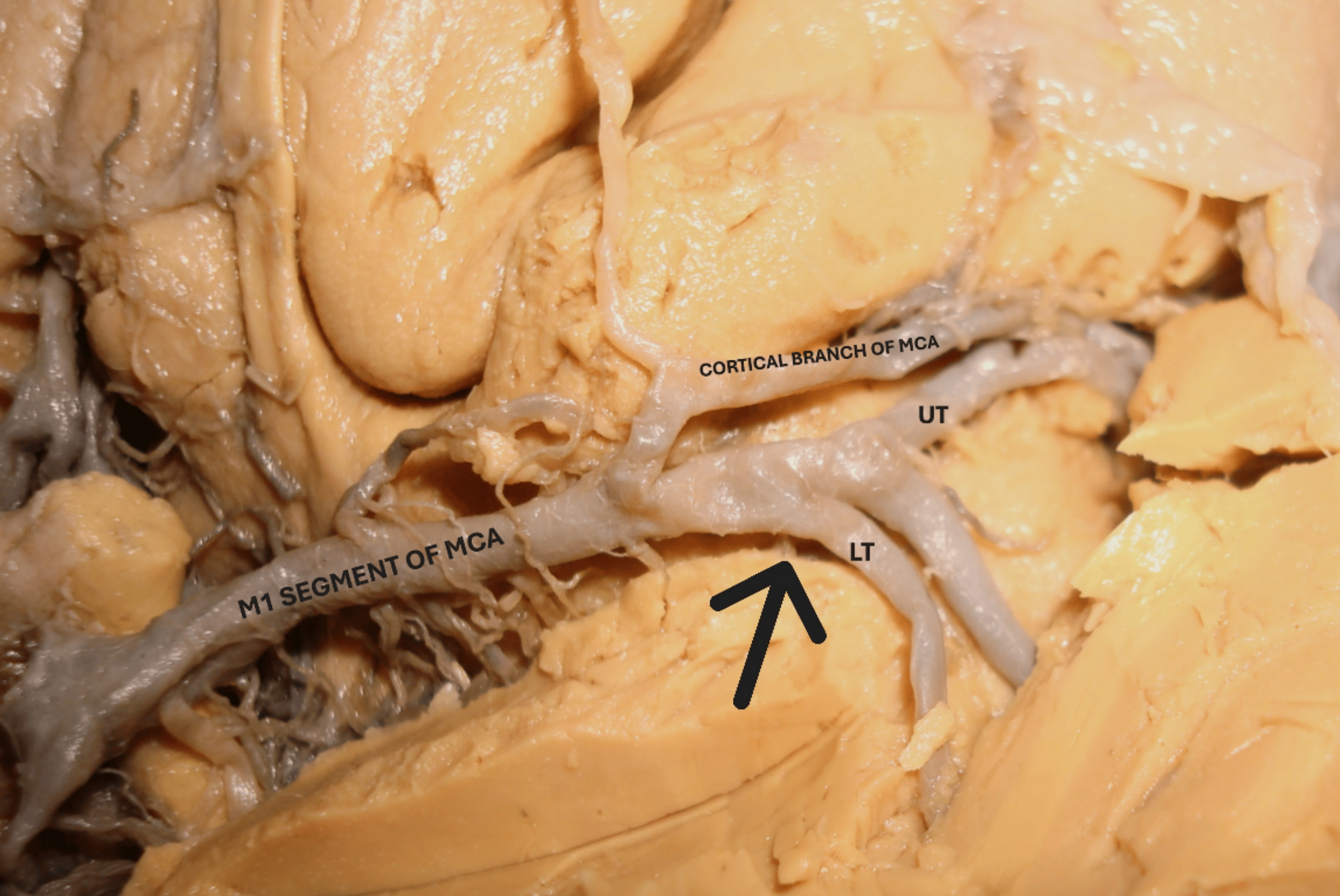
Basal surface of the brain showing the division of M1 segment into pseudo-trifurcation. The main trunk of M1 split into two primary divisions (UT and LT), but one division immediately branched off from UT, creating the illusion of trifurcation of the trunk. The black arrow indicates the site of pseudo-trifurcation. MCA: middle cerebral artery; UT: upper trunk; LT: lower trunk

**Figure 5:**
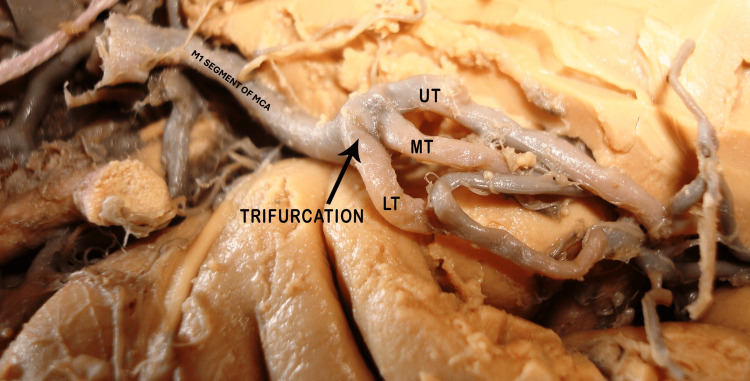
MCA trifurcates into UT, MT, and LT. The black arrow indicates the site of trifurcation. MCA: middle cerebral artery; UT: upper trunk; MT: middle trunk; LT: lower trunk

A pattern of division of the MT of the M1 segment was also observed in this study. It was classified as true bifurcation (MT of M1 divided into two primary divisions) (Figure 3), pseudo-trifurcation (MT of M1 split into two primary divisions, but one division immediately branched off, creating the illusion of trifurcation) (Figure 4), and true trifurcation (where the MT is distinctly divided into three divisions at a single point) (Figure 5 and Table 2).

**Table 2:** Division patterns of the main trunk of the middle cerebral artery (MCA).

Pattern of division	No. of cases (%)
Early bifurcation	2 (6.6)
Bifurcation	23 (76.7)
Pseudotrifurcation	3 (10)
True-trifurcation	2 (6.6)

**Figure 6:**
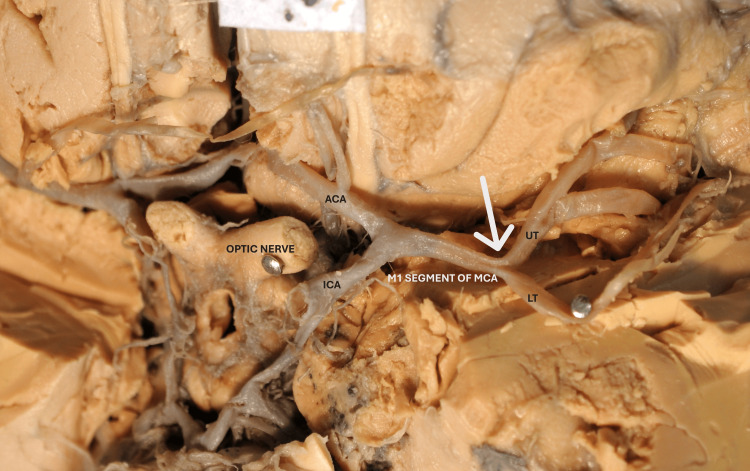
Basal surface of the brain showing the early bifurcation of the M1 segment of MCA into UT and LT. The white arrow indicates the exact site of bifurcation. MCA: middle cerebral artery; ACA: anterior cerebral artery; ICA: internal carotid artery; UT: upper trunk; LT: lower trunk

Early bifurcation was referred to as bifurcation taking place within 10 mm of the MCA origin (Figure 6).

**Figure 7:**
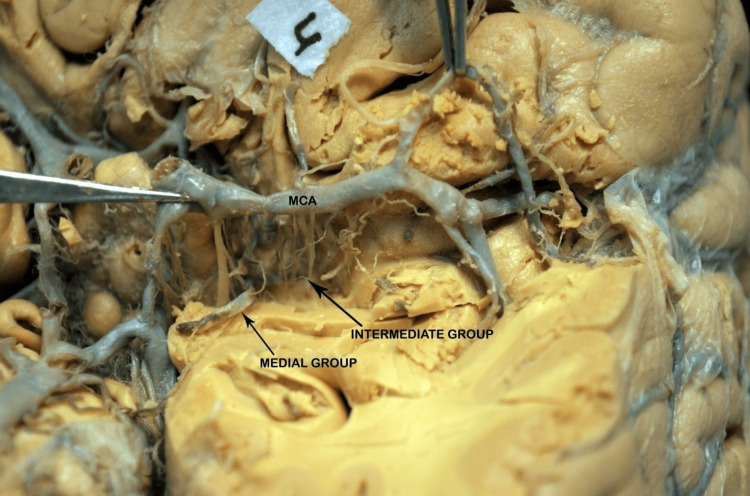
Basal surface of the brain showing perforators arising from the M1 segment of the MCA. The black arrows indicate the medial and intermediate groups of perforators. The medial group arises from the proximal M1 segment, whereas the intermediate group arises from the midportion of the M1 segment. MCA: middle cerebral artery

During the examination of perforators, special attention was paid to their number and origin. To describe the origin of the perforators, we divided the total length of the M1 segment into three parts: medial 1/3, intermediate 1/3, and lateral 1/3. Perforators from the M1 segment were divided into three groups: medial, intermediate, and lateral (Figure 7).

**Figure 8:**
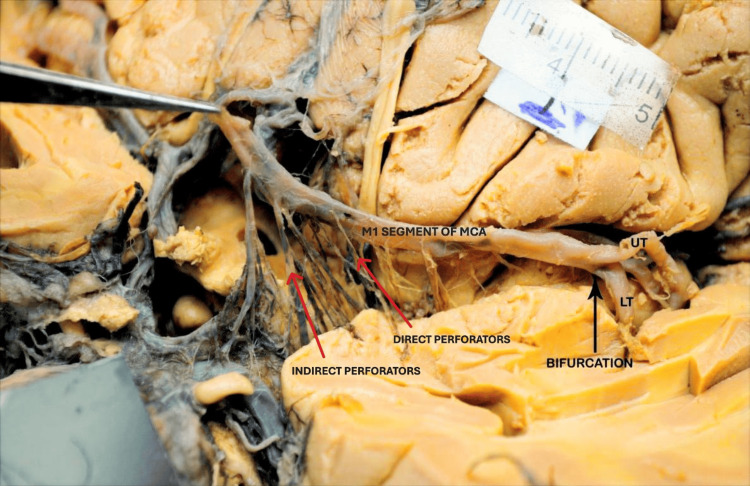
M1 segment of the MCA showing the origin of direct and indirect perforators. The red arrows indicate the direct and indirect perforators, and the black arrow indicates the bifurcation site of the M1 segment. MCA: middle cerebral artery; UT: upper trunk; LT: lower trunk

**Figure 9:**
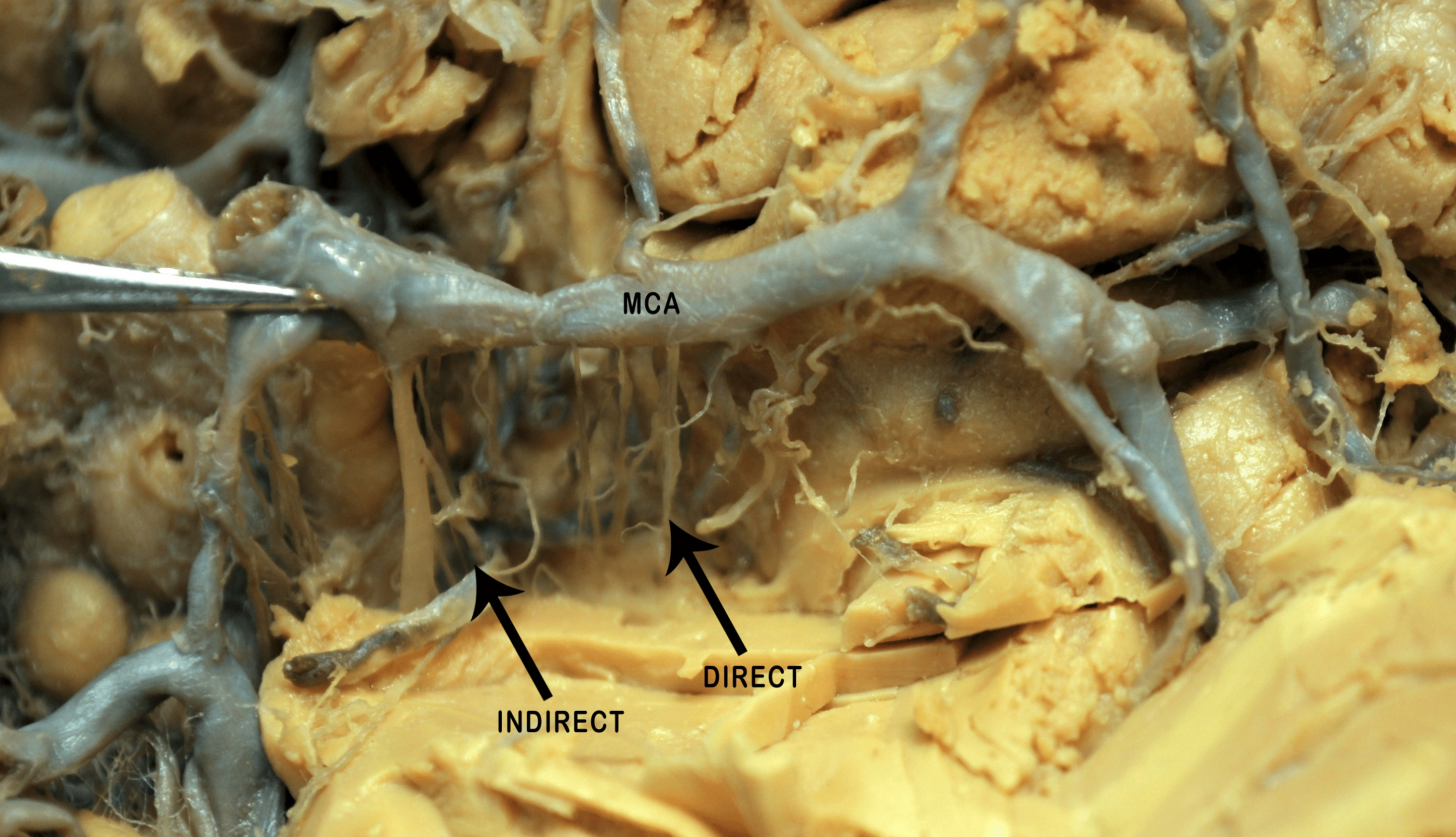
M1 segment showing indirect and direct perforators arising from the MCA trunk (black arrows). MCA: middle cerebral artery

Perforators were also grouped into direct and indirect categories. Direct perforators originate as a single trunk and then penetrate the brain substance as a single trunk without any division in 17 (56%) cerebral hemispheres, whereas indirect perforators emerge as a single trunk but bifurcate into several branches prior to entering the brain’s substance in 13 (44%) cerebral hemispheres (Figures 8, 9).

In the medial group, the average number of perforators arising from the group was 3 and ranged from 0 to 8. In the intermediate group, the average number of perforators arising from the group was 4 and ranged from 2 to 7. In the lateral group, the average number of perforators arising from the group was 2 and ranged from 0 to 8 (Table 3).

**Table 3:** Distribution of perforators arising from the M1 segment of the MCA (direct and indirect). MCA: middle cerebral artery

Perforators	Medial direct	Medial indirect	Intermediate direct	Intermediate indirect	Lateral direct	Lateral indirect
Range	0-8	0-7	2-7	2-7	0-8	0-2
Average	3	2	4	2	2	1

After bifurcation, the trunk of the M1 segment was divided into the upper and lower divisions, and the trifurcated trunk was divided into the upper, middle, and lower divisions. In the present study, all the post-bifurcation perforators arose from the upper division of the M1 segment.

**Figure 10:**
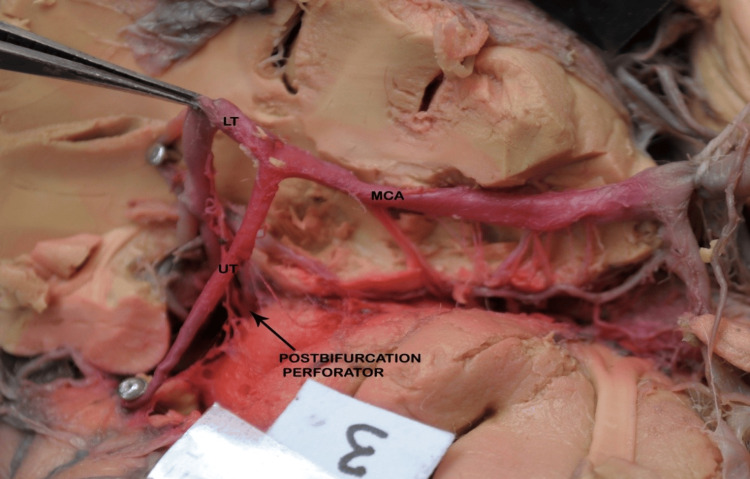
Basal surface of the brain showing post-bifurcation perforators arising from the UT of the MCA. No perforators are seen arising from LT. The black arrow indicates the perforators. MCA: middle cerebral artery; UT: upper trunk; LT: lower trunk

Perforators also arise from the post-bifurcation trunk of the MCA (Figure 10). The total number of perforators arising from the post-bifurcation trunk was 47, of which 27 (57%) arose as a single trunk (direct) and 20 (43%) were branched (indirect).

## Discussion

The MCA is considered the most significant vessel that supplies blood to major cortical areas and deep brain structures [1,2]. According to the classical description of the MCA in the literature, it is divided into four segments: the M1 segment extends between the origin of the MCA and the genu of the insula; M2 runs from the limen insula to the circular sulcus of the insula; and the M3 segment continues from the circular sulcus to the Sylvian fissure surface. The M4 segment proceeds from the Sylvian fissure to the branches in the lateral convexity [10,11]. Further refinement of this classification was proposed by Türe et al., who identified an additional fifth segment and mentioned the anatomical landmarks for segment demarcation [12]. Türe et al. described an additional fifth segment, where M4 and M5 correspond to the parainsular and terminal segments, respectively, and identified MCA bifurcation as a demarcation line between the M1 and M2 segments of the MCA [13,14].

In the present study, the diameter of the M1 segment at the origin was recorded as 2.35 ±0.51 mm, with no significant difference between the two cerebral hemispheres. These findings align closely with those of previous reports, confirming the consistency of MCA dimensions across different populations. The mean length of the M1 segment was 17.13 ± 2.8 mm (right hemisphere) and 16.98 ± 2.4 mm (left hemisphere), consistent with the earlier reported finding of 15 ± 1.3 mm [15], 20 mm [16], and 14-16 mm [17,18]. In previous studies, the mean diameter of M1 was 3 ± 0.1 mm [16] and 2-4 mm [17].

Bifurcation of the M1 segment of the MCA was observed in 23 (76%) of the cases in the present study, similar to that previously reported in other studies [5,6], and trifurcation in two (6%) of cases. In the present study, the pseudo-trifurcation was observed in three (10%) of cases, and all these variants were lying on the right side of the cerebral hemispheres. At the pseudo-trifurcation, the MT of M1 split into two primary divisions, but one division immediately branched off, creating the illusion of trifurcation of the trunk. In the present study, we did not encounter a single trunk of MCA that continues as only a trunk, and all the branches are from a single trunk. Comparatively, regional studies have reported some variation in these branching patterns, highlighting potential population-specific differences. A North Indian study [19] reported bifurcation in 64%, single trunk in 6%, and trifurcation in 29% of cases. Another variant, a twig-like middle cerebral artery (tMCA), was also reported as an uncommon congenital vascular anomaly that can resemble vaso-occlusive conditions in advanced imaging studies [20]. Therefore, its recognition is critical to avoid misdiagnosis and inappropriate management.

The division of the MCA is a frequent site for cerebral aneurysm development, posing considerable difficulties for surgical treatment [21]. One such challenge is early bifurcation of the MCA, which can complicate surgical planning and intervention. A bifurcation occurring 10 mm away from the MCA origin was considered an early bifurcation, which was reported in two (6.6%) cases in the present study. James et al. [9] described the same in 3% of the cases. Duplication of the MCA is an uncommon anatomical variation where an abnormal MCA branches from the far end of the ICA. This variant has important clinical implications, especially when occluded, because it may not be readily apparent on routine imaging. When blocked, a duplicated MCA can lead to severe impairments similar to those caused by M2 vessel blockage, yet it may not show clear signs on vessel imaging through computed tomography angiography (CTA) or magnetic resonance angiography [22].

The majority of saccular aneurysms in the MCA are reported close to the primary arterial division/bifurcation [18,23]. Surgeons must therefore remain vigilant for perforating branches during aneurysm dissection to avoid inadvertent injury. Thus, during aneurysm dissection, the surgeon should recognize the perforating branches that may occasionally arise from the secondary trunk and take a recurrent course. Aneurysms lying close to the MCA bifurcation may present symptoms similar to those of ischemic stroke in the internal capsule due to compression or distortion of the perforating artery near the bifurcation site [24]. LSAs originate from the MCA. These small perforators supply vital subcortical structures, making their preservation crucial during surgical interventions. These small perforating arteries supply blood to critical subcortical regions, including the basal ganglia and posterior limb of the internal capsule [23-25]. Large thrombosed aneurysms pose significant challenges in both diagnosis and treatment due to their instability and elevated risk of ischemic stroke. Despite advances in endovascular techniques, surgical clipping remains the mainstay treatment for certain aneurysm types, particularly saccular aneurysms in the anterior circulation. Surgical clipping is still frequently used to treat intracranial aneurysms, particularly saccular aneurysms in the anterior circulation [26].

Consistent with earlier studies [16,23], the number of perforators in various groups (including medial, intermediate, and lateral) ranged from 0 to 9, with a maximum average of four in the intermediate direct group of perforators. Our findings corroborate earlier observations regarding the origin and branching patterns of these perforators, which are integral to neurosurgical planning. Oo et al. [27] described in a study that all perforators (100%) formed as a single branch and did not mention any division from the perforators. In the present study, we observed both types of perforators with the single trunk and multiple branches. The location of origin of these perforating arteries is crucial for planning neurosurgical procedures.

The close proximity to perforators and early cortical branches makes operating on prebifurcation MCA aneurysms particularly challenging. This anatomical complexity demands meticulous surgical techniques to preserve vital vessels. Giant aneurysms located at the wide-necked M1 segment can be managed by clipping the neck in a manner parallel to the M1 segment. It is important to position the clip away from the neck to ensure that the closure line leaves a portion of the neck intact, which helps prevent any compromise of the parent artery [28]. In our study, 88% of the perforating arteries arose before bifurcation, pre-bifurcation trunk of M1, and 12% arose from secondary trunks (superior trunk, 10%; inferior trunk, 2%). These proportions align with those reported in the literature, although the total number of perforators observed varies across studies. In the literature, the number of perforators reported ranges from 3 to 15 [26]. In the present study, the number of perforators ranged from 0 to 9. Two patterns of origin of the perforators were observed. Perforators were grouped into direct and indirect categories. Direct perforators originate as a single trunk and then penetrate the brain substance as a single trunk without any division, observed in 56%, whereas indirect perforators emerge as a single trunk but bifurcate into several branches prior to entering the brain’s substance, observed in 44% of cases. Perforators were also grouped into medial, intermediate, and lateral groups depending upon their emergence from the M1 trunk. In this study, the intermediate group was the most consistently observed group and appeared in all the specimens.

The medial group was observed in 28 of the 30 studied hemispheres. Its typical origin and course are important landmarks during surgical approaches. Predominantly, they emerged as a single twig that emerged directly from the M1 segment, positioned 3-7 mm away from the MCA origin, and proceeded in a nearly perpendicular direction to the anterior perforated substance (APS). The intermediate group, which was the most consistently observed, began as a single stem artery from the M1 trunk. This artery then splits into two to three branches at a distance of 10-14 mm from the MCA origin, following an oblique path to the APS. The lateral group, originating from either the MT or secondary trunks (mainly the superior trunk), was identified in 20 out of the 30 hemispheres, located 13-16 mm from its origin. In 6.6% of the cases in which early bifurcation was found, the medial group of perforators was absent, and the number of perforators arising from the intermediate group was higher (ranging from four to nine) than that of the rest of the specimens (ranging from two to seven). This observation suggests compensatory vascular patterns in response to the anatomical variations. It was also observed that in these cases, the post-bifurcation trunk always gave rise to the perforators. Occlusion or stenosis of the LSA results in small infarcts or lacunar strokes, which must be differentiated from large-artery atherosclerosis in the etiological diagnosis of stroke [3,4]. Therefore, a detailed evaluation of LSAs is critical for accurate stroke diagnosis and management. Consequently, evaluating the structure and function of LSAs holds significant clinical importance.

Many studies have reported that the more proximal the division of the M1 segment into the bifurcation, the greater the chances of perforators arising from the secondary trunks. Our findings support this correlation, emphasizing the need to consider the bifurcation location during surgical planning. In the present study, where M1 bifurcated into secondary divisions, perforators arose from the upper secondary trunk. It has also been observed that in those cases where the length of the M1 segment (pre-bifurcation length) was 10 mm (6.6% of cases), the number of perforators was densely placed near the bifurcation and most commonly came from the intermediate group, and also the post-bifurcation trunk observed in the present study. For the effective surgical management of aneurysms, it is crucial to carefully isolate perforators, avoid vessel kinking, and choose the most suitable clip based on the MCA’s anatomy [25]. Incorporating cadaveric dissection of the cerebral vasculature into the neurosurgical curriculum is highly beneficial to enhance surgical training and outcomes. Incorporating cadaveric dissection of cerebral vasculature into the neurosurgical training curriculum would enable resident doctors to grasp the intricate neuroanatomy of the brain vasculature and build confidence during surgical procedures [8,9].

MCA embryological origin is closely associated with the development of the cerebral hemispheres of the developing brain [29]. Initially, it appears that the plexiform network arises from the trunk of the ICA detected at the fourth week of intrauterine life. Along with further development, the twig initially arose from the cranial division of the ICA from the MT of the MCA [30]. Failure of segmental fusion may lead to the appearance of early bifurcation, trifurcation, a single trunk, and another congenial numerical and morphological variations of MCA [30].

Understanding the anatomy and the susceptibility of an early branch is crucial from a surgical point of view, as it may create a false appearance of bifurcation and result in misinterpretation of the true bifurcation and MCA branches. Identifying these anatomical details may help the surgeon to repudiate intraoperative errors.

Despite providing valuable insights into the morphology of the M1 segment of the MCA, this study has certain limitations. The relatively small sample size of 30 formalin-fixed cerebral hemispheres may not fully represent the range of anatomical variations in the general population. Additionally, variability in the identification and counting of small perforating branches may influence comparisons with previous studies. Future research involving larger, multiethnic samples and radiological correlation in living subjects would enhance the generalizability and clinical relevance of these observations.

## Conclusions

The detailed anatomical characterization of the MCA and its perforating branches underscores their critical relevance in neurosurgical and endovascular interventions. Variations in segmental divisions, bifurcation patterns, and perforator origins necessitate careful preoperative planning to avoid complications such as vessel kinking, ischemic stroke, or neurological deficits. Recognition of early branches and precise identification of perforators, particularly the LSAs, are essential for successful aneurysm management and stroke differentiation. Integrating cadaveric dissection into surgical training enhances the understanding of MCA microanatomy, ultimately improving clinical outcomes through meticulous operative technique and informed decision-making.
